# Binding of *cis*-[Ru(phen)_2_(3,4Apy)_2_]^2+^ to Model Lipid Membranes: Implications for New Tools in the Development of Antiamyloid Drugs

**DOI:** 10.1021/acs.langmuir.4c03552

**Published:** 2024-12-17

**Authors:** Maria Laura da Cruz Garcia, Rafaela Ribeiro Paixão, Wallance M. Pazin, Osvaldo N. Oliveira, Paul S. Cremer, Rose Maria Carlos

**Affiliations:** † Department of Chemistry, Federal University of São Carlos, CP 676, CEP, São Carlos, São Paulo 13565-905, Brazil; ‡ Department of Physics and Metereology, São Paulo State University, CEP, Bauru, São Paulo 17033-360, Brazil; § Sao Carlos Institute of Physics, University of Sao Paulo, CP 369, CEP, São Carlos, São Paulo 13560-970, Brazil; ∥ Department of Chemistry and Department of Biochemistry and Molecular Cell Biology, The Pennsylvania State University, University Park, Pennsylvania 16802, United States

## Abstract

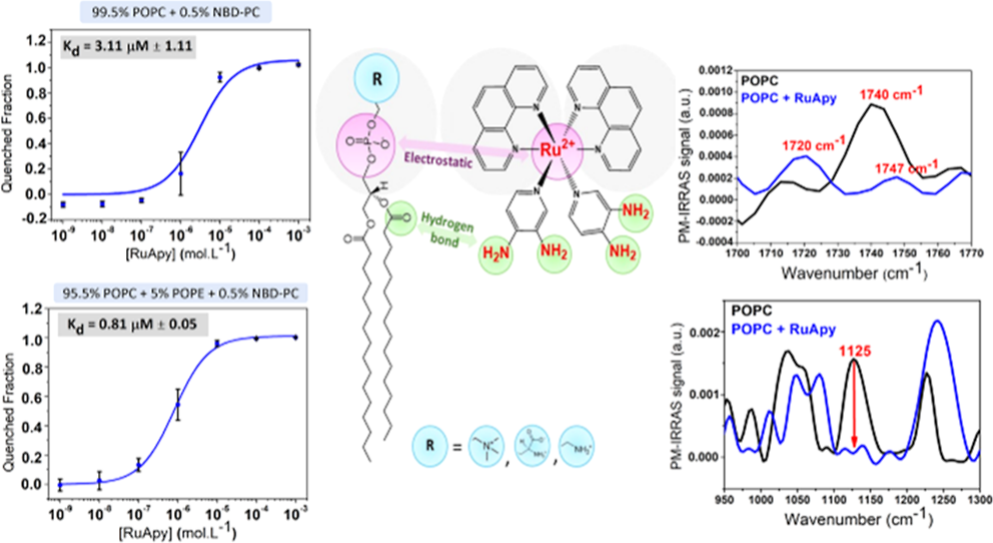

This study explores the interactions of the *cis*-[Ru(phen)_2_(Apy)_2_]^2+^ complex (RuApy, phen = 1,10-phenanthroline, Apy = 3,4-aminopyridine) with model lipid membranes to explain the role this complex plays in mitigating Aβ toxicity in PC12 neuronal cells. Fluorescence quenching, surface pressure isotherms in Langmuir monolayers, and infrared reflection–absorption analyses revealed that the positively charged RuApy interacts with the phosphate headgroups of monolayers, indirectly affecting ester carbonyl groups through hydrogen bonding with the amino group of the pyridine ligand of RuApy. These results offer a scenario for the protective effect of RuApy against Aβ toxicity in neuronal cells in which these interactions shield the electrostatic interactions of Aβ with lipid membranes, preserving membrane integrity and mitigating the deleterious influence of Aβ. This opens new avenues for antiamyloid strategies, focusing on compounds that prevent salt-bridge formation between bilayer membranes and amyloid proteins, aiding in the rational design of effective antiamyloid agents for therapeutic application.

## Introduction

Cell membranes are crucial in mammalian physiological processes as they mediate communication and signaling between cells and control the entry and exiting of ions, small molecules, and other ligands.^1^ Any change in composition that impacts the integrity and structure of the membrane affects cell functionality, with consequences for both healthy and pathological organisms.^2^ Therefore, studies devoted to monitoring changes in the shape, morphology, permeability, and components of lipid membranes are important for the development of new drug molecules and for disease diagnosis. For instance, drug-lipid membrane interactions have been studied to evaluate the potential toxicity of ibuprofen, a nonsteroidal anti-inflammatory drug used for pain relief and reduction of inflammation and fever.^3,4^ Sun et al. showed that ibuprofen interacts with lipid membranes through electrostatic and H-bonding interactions in the micromolar concentration range but then embeds itself within the membrane and has a detergent-like effect as the concentration is raised further.^5^ Lee et al. showed that ibuprofen affects the water permeability of phosphatidylcholine (PC) membranes with saturated chains in the presence of cholesterol.^6^

Synaptic loss and neuronal cell death in Alzheimer’s disease are believed to be linked to the formation of membrane pores in the presence of beta amyloid peptide (Aβ).^7^ It has also been reported that Aβ interacts with negatively charged lipid membranes and increases the formation of toxic β-sheet structures of Aβ. A putative molecular mechanism involves the binding of positively charged residues on Aβ to the negatively charged phosphate headgroups of the membrane surface, which in turn drives the nonpolar residues of Aβ into the bilayer to form hydrophobic interactions with the acyl chains on the phospholipids. These interactions induce membrane rupture and, consequently, lead to the loss of cellular homeostasis and cell death.^8,9^ It has been demonstrated that Ca^2+^ ions interact with the negatively charged phosphate groups linked to the headgroups of lipid membranes, reducing the interactions between Aβ and lipid membranes.^10^ In fact, these results indicate that the development of new drug strategies that are able to shield the Aβ interactions from the cell membrane would benefit from a more comprehensive understanding of the toxic mechanism of Aβ related to cell membrane rupture and serve as a tool for the discovery of new treatments for AD.

**Scheme 1:**
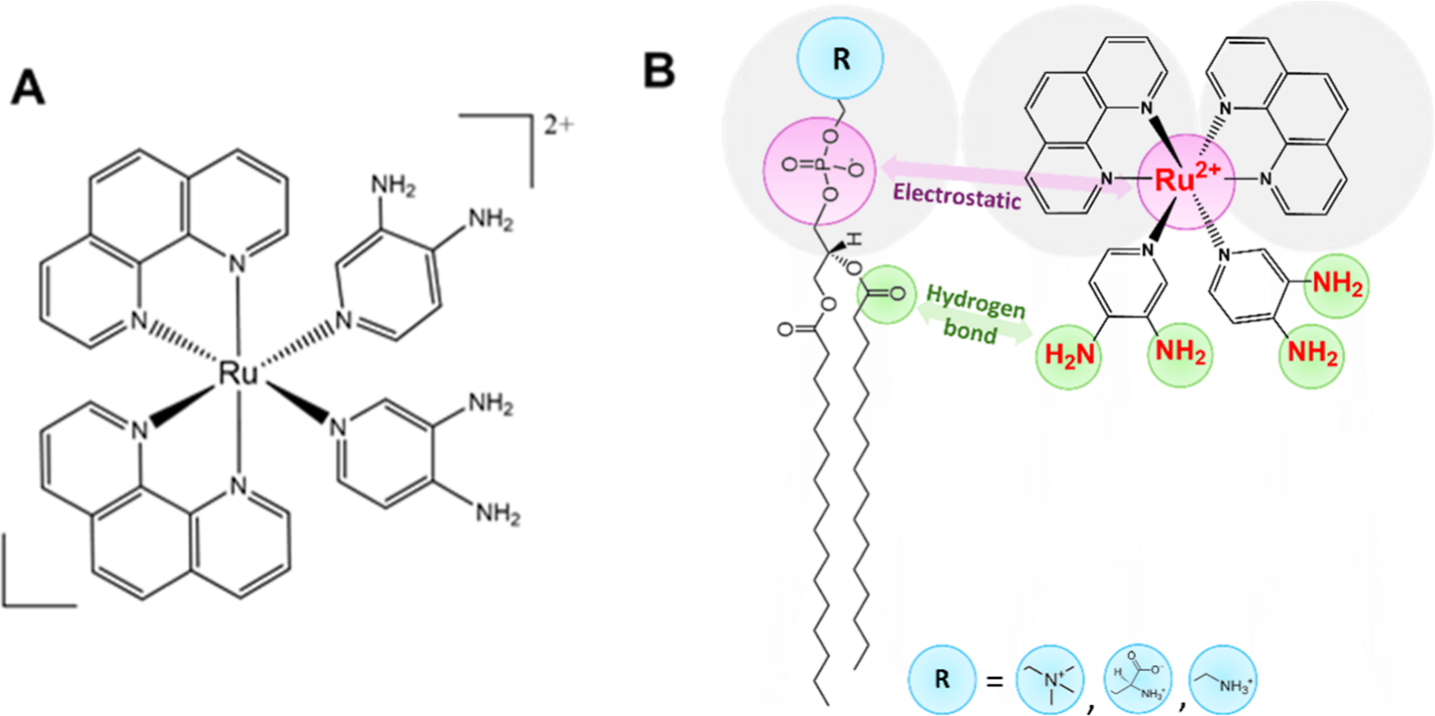
(A) Molecular Structure of RuApy Complex and (B) Possible Interactions of RuApy to Lipids

In this context, our group has demonstrated that the water-soluble and luminescent complex *cis*-[Ru(phen)_2_(Apy)_2_]^2+^ (RuApy, Apy = 3,4-aminopyridine, phen = 1,10-phenanthroline) enables the mapping of the Aβ aggregation process through the complex’s luminescence response. Significantly, RuApy protects PC12 cells against the toxicity of Aβ aggregation by shunting the process down to a nontoxic pathway.^11−13^ RuApy accumulates in the cytoplasmatic region of the NEURO2A cells without apparent toxicity. This potentially makes the RuApy complex a promising theranostic candidate against diseases associated with amyloid proteins. Since Aβ targets the cell membrane through electrostatic and H-bonding interactions, it is important to understand the interactions of RuApy with lipid membranes, which may, in turn, alter the way Aβ interacts with lipids. RuApy contains both a hydrophobic and a hydrophilic region around the positively charged metal ion complex, Scheme 1A. The interactions of this complex with phospholipids can occur either with the polar head groups or with the hydrophobic acyl chains. Such interactions may affect the physical and chemical properties of the membrane, especially in the presence of cholesterol.^14,15^ Indeed, changes in the lipid membrane due to RuApy interactions may lead to direct or indirect consequences for Aβ toxicity. These putative effects motivated us to explore RuApy–lipid membrane interactions in detail.

Herein, the interactions of RuApy with neutral PC and negatively charged lipid membranes achieved by incorporating phosphatidylserine (PS)—a lipid with a net negative charge at physiological pH—were explored in supported lipid bilayer (SLB) platforms using fluorescence quenching assays. RuApy–lipid interactions were also studied in lipid monolayers using pressure (π)–area (*A*) isotherm measurements as well as infrared reflection–absorption spectroscopy (PM-IRRAS). The results reveal the substantial role that both electrostatics and H-bonding play in the interactions between the coordination complex and the membrane (Scheme 1B). Moreover, they may even suggest explanations for the protective effect of RuApy with PC12 cells against toxic species associated with Aβ oligomers.

## Results and Discussion

**Scheme 2:**
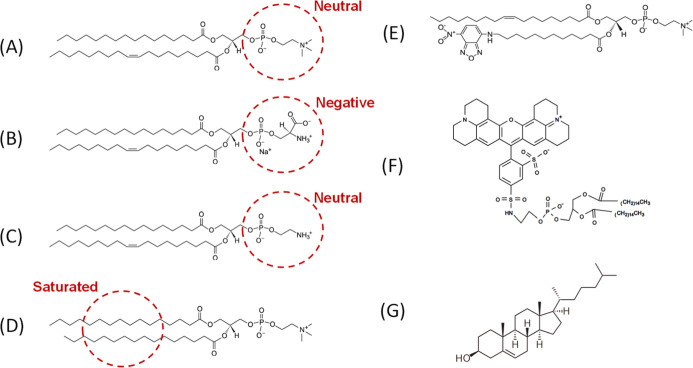
Molecular Structure of Lipids: (A) POPC, (B) POPS, (C) POPE, (D) DPPC, (E) NBD-PC, (F) TR-DHPE, and (G) Cholesterol

The structures of all lipids and dyes used in the studies of the interaction between RuApy and the membrane model system are shown in Scheme 2.

### Fluorescence Binding Assays for RuApy–SLB Interactions

**Figure 1:**
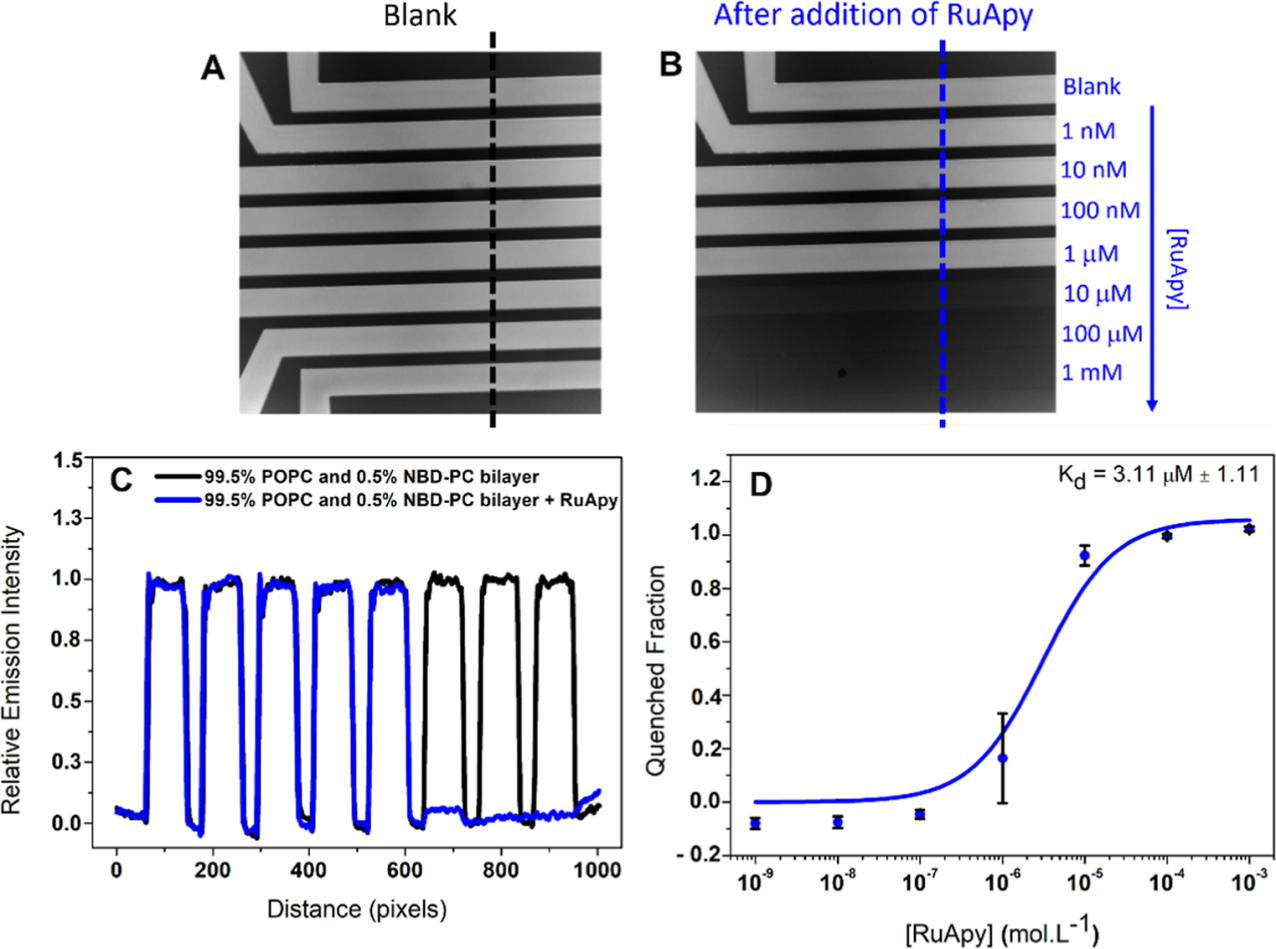
Epifluorescence images of SLBs composed of 99.5% POPC and 0.5% NBD-PC in an eight-channel microfluidic device (A) in the absence of RuApy and (B) after the introduction of RuApy at the concentrations indicated. (C) Fluorescence line profiles across the microfluidic channels before (black) and after (blue) the introduction of RuApy into the channels. (D) Data was plotted to determine *K*_d_ by fitting to eq 2.

The interactions between RuApy and SLBs were investigated by a fluorescence assay using a dye-labeled lipid incorporated into SLBs formed in microfluidic channels. The first experiment was performed using bilayers composed of 99.5 mol % 1-palmitoyl-2-oleoyl-*sn*-glycero-3-phosphocholine (POPC) with 0.5 mol % 1-oleoyl-2-[12-[(7-nitro-2–1,3-benzoxadiazol-4-il)amino]dodecanoyl]-*sn*-glycero-3-phosphocholine (NBD-PC). To determine the equilibrium dissociation constant, *K*_d_, a blank measurement was first made after excess vesicles were washed away, which led to uniform fluorescence intensity from the nascent bilayer film (Figure 1A). At this point, different concentrations of RuApy were flowed into each channel over a concentration range from 0 to 1 mM for 1 h to reach equilibrium (Figure 1B). This led to nearly complete quenching of the NBD-PC at higher RuApy concentrations, as the luminescence response reached a saturation plateau. The fluorescence intensity as a function of position along the black and blue dashed lines in Figure 1A,B, respectively, was used to plot the line profiles for the eight different RuApy concentrations that were employed (Figure 1C). The values from the eight different concentrations can then be used to construct the plot shown in Figure 1D. Moreover, the data could be fitted to eq 2 to obtain *K*_d_. Fitting these data, the binding curve for the RuApy/SLBs yielded *K*_d_ = 3.11 μM ± 1.11.

One potential concern with the experiments performed in Figure 1 is that the RuApy complex may interact only with the NBD dye molecules instead of the PC lipids. As such, a control experiment was performed with membranes composed of 99.7 mol % POPC and 0.3 mol % TR-DHPE (λ_exc_ = 595 nm and λ_em_ = 615 nm). In this experiment, the RuApy concentration varied from 0 to 300 μM. The dye moiety was substantially larger and positioned on the head groups of the phospholipids. Therefore, one might anticipate that it would decrease (i.e., tighten) *K*_d_ if RuApy–dye interactions were significant. Nevertheless, this measurement led to a *K*_d_ value of 3.60 μM ± 0.40 (Figure S2), which was the same within experimental error as the value obtained with NBD-PC. Therefore, the control measurement is consistent with the hypothesis that RuApy–POPC interactions (rather than RuApy–dye interactions) are overwhelmingly responsible for the measured *K*_d_ values.^18,19^

### Mechanism of RuApy–Membrane Interactions (Electrostatics and H-Bonding)

The affinity between RuApy and POPC may result from hydrogen bonds between the PO^2–^ and CO groups on the phospholipids and the NH_2_ group of the 3,4Apy ligand. Because RuApy is positively charged, it may interact electrostatically with the phosphate moiety on zwitterionic PC lipids. However, the incorporation of PS, a lipid with a net negative charge, is an effective way to enhance electrostatic interaction between the membrane and RuApy. To this end, SLBs were prepared with 94.5 mol % POPC, 5 mol % 1-palmitoyl-2-oleoyl-*sn*-glycero-3-phospo-l-serine (POPS), and 0.5 mol % NBD-PC. In this case, microfluidic experiments revealed that *K*_d_ decreased only slightly to 1.99 μM ± 0.29 (Figure S3). This result indicates that electrostatic interactions play a modest role in the interactions between RuApy and lipid membranes, or these interactions were already screened in the buffer [phosphate-buffered saline (PBS), 137 mM NaCl].

**Figure 2:**
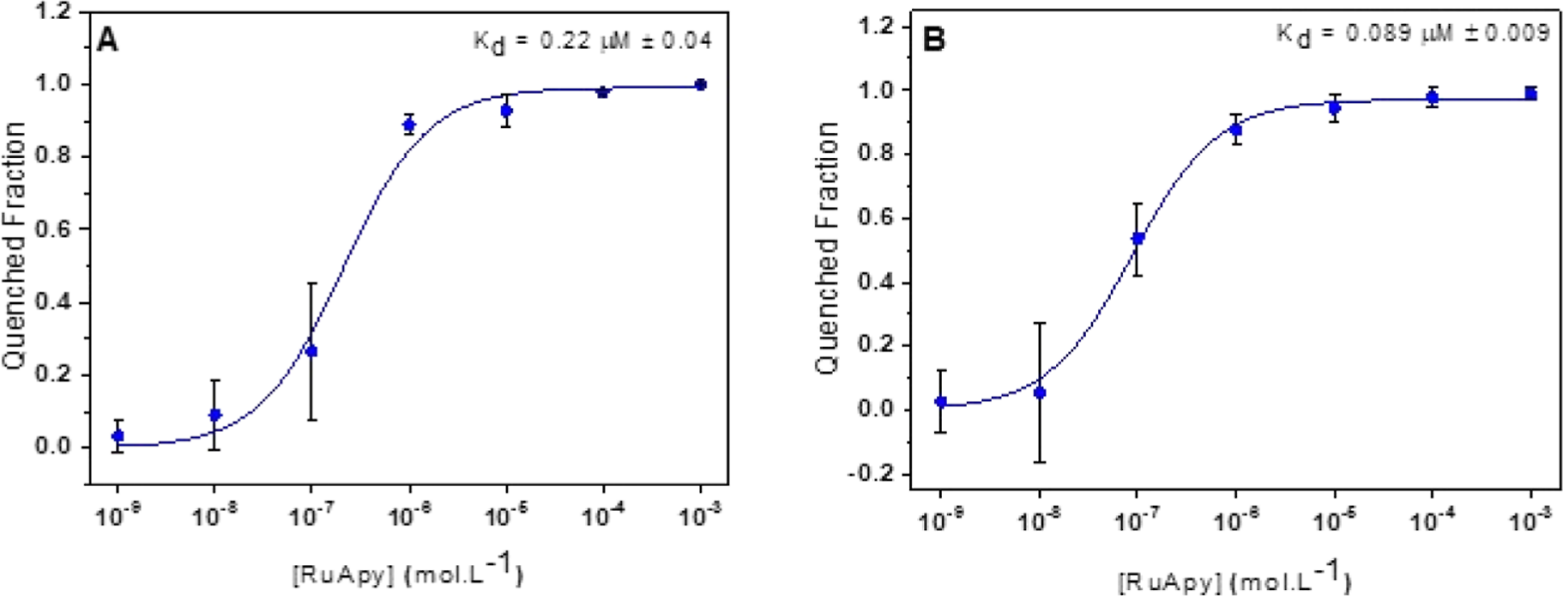
*K*_d_ for RuApy–SLB interactions in 10 mM phosphate buffer at pH 7.4 in the absence of NaCl. The bilayers consisted of (A) 99.5% POPC and 0.5% NBD-PC and (B) 94.5% POPC, 5% POPS, and 0.5% NBD-PC.

To address the buffer screening issue, binding experiments were repeated with 10 mM phosphate buffer but without any NaCl. Two bilayer conditions were probed with 99.5 mol % POPC and 0.5 mol % NBD-PC (Figure 2A) as well as with 94.5 mol % POPC, 5.0 mol % POPS, and 0.5 mol % NBD-PC (Figure 2B). The corresponding *K*_d_ values were 0.22 μM ± 0.04 and 0.089 μM ± 0.009, respectively, which represented tightening by a factor of 14 in the absence of negatively charged lipids and a factor of 23 in the presence of 5 mol % POPS. Analogous experiments with TR-DHPE also showed that *K*_d_ tightened in the absence of 137 mM NaCl (Figure S4). Thus, the results are consistent with electrostatic interactions being significant in RuApy–lipid membrane interactions, particularly in bilayers containing negatively charged lipids like POPS. Indeed, this should be expected, as bilayers with a net negative charge experience enhanced electrostatic interactions due to the increase in the Debye length in the absence of NaCl.^16,17,22−24^

**Figure 3:**
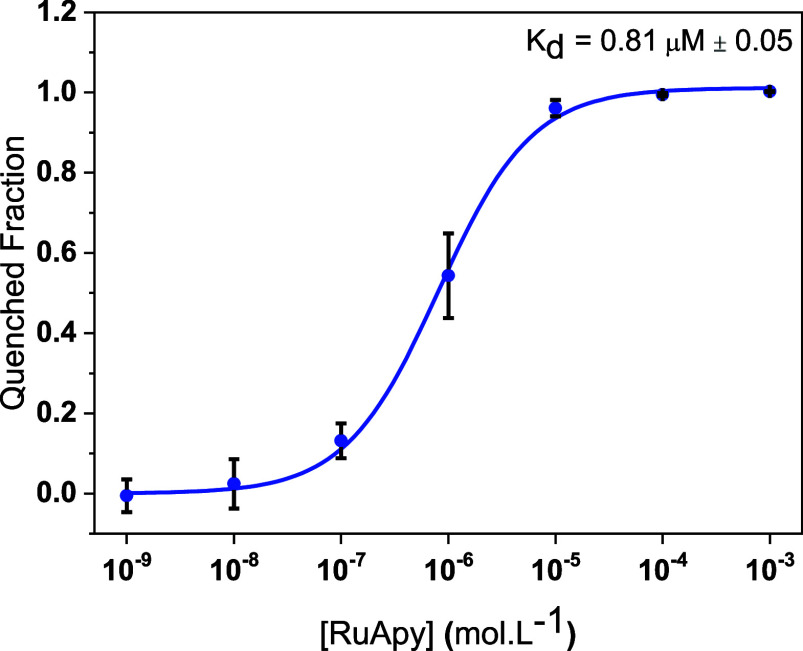
Fluorescence quenching curve obtained for RuApy/SLB interactions in PBS buffer with 137 mM NaCl at pH 7.4. The bilayers were composed of 94.5% POPC, 5% POPE, and 0.5% NBD-PC.

In addition to electrostatic effects, H-bonding between RuApy and the lipid membranes was explored. This was done by introducing 1-palmitoyl-2-oleoyl-*sn*-glycero-3-phosphoethanolamine (POPE) lipids into the SLBs. The key difference between POPE and POPC is that the methyl groups on the choline moiety of PC lipids are replaced with hydrogen atoms (Scheme 2C).^18,19^ This allows H-bond donation to take place. Experiments were run in membranes with 94.5 mol % POPC, 5.0 mol % POPE, and 0.5 mol % NBD-PC. Figure 3 shows a decreased *K*_d_ of 0.81 μM ± 0.05, nearly a factor of 4 tighter than when just POPC lipids were employed (Figure 1D). This result suggests that hydrogen bonding can indeed play a role in RuApy–bilayer interactions.

The enhanced interaction between RuApy and the membrane when POPE is introduced agrees with studies describing interactions between POPE and POPC within bilayers.^18−23^ These studies propose a decrease in the area occupied per POPC due to hydrogen bonding between POPE and POPC, which relieves electrostatic repulsion between POPC headgroups.^20^ For instance, an increase in POPC/tetracaine affinity was attributed to hydrogen bonding between the R-NH_3_^+^ group of POPE and the C=O group of tetracaine because this interaction relieved the lipid packing constraints.^24,25^ Evidence of hydrogen bonds between the donor NH group of tryptophan and the carbonyl groups on the lipids was obtained with various techniques, including Raman spectroscopy.^26^ Pervaiz and co-workers demonstrated that hydrogen bonding between the oxygen atom from Ser122 on the acetylcholinesterase enzyme (AChE) and the NH groups of the imidazole is responsible for the inhibitory effect in a series of imidazole compounds against AChE activity.^26,27^ Similar behavior may be expected in the RuApy/lipid membrane systems investigated here because the donor NH_2_ groups of the 3,4Apy ligands can form analogous hydrogen bonds.^28^

**Figure 4:**
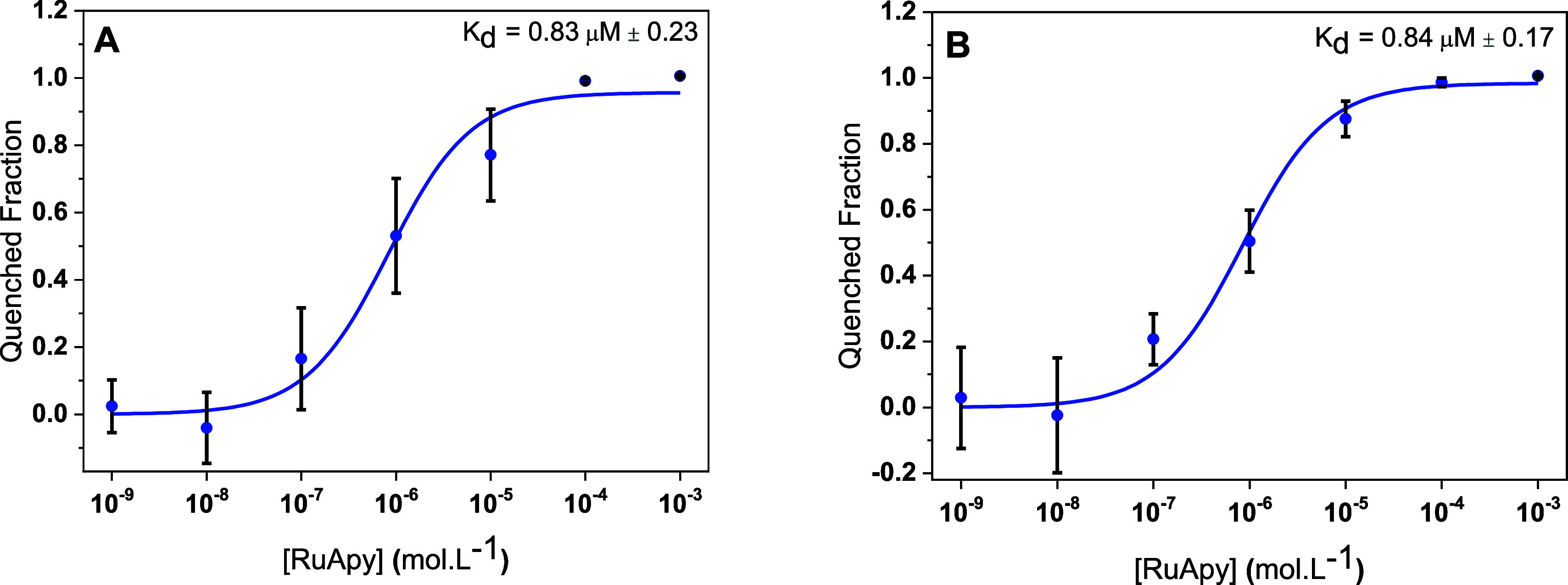
Fluorescence quenching curves to determine *K*_d_ for RuApy/SLB interactions in PBS buffer pH 7.4 in D_2_O with bilayers composed of (A) 99.5% POPC and 0.5% NBD-PC and (B) 94.5% POPC, 5% POPS, and 0.5% NBD-PC.

To further explore the influence of hydrogen bonding on RuApy–lipid interactions, binding affinity experiments were performed in PBS buffer solutions with 137 mM NaCl prepared in D_2_O instead of H_2_O. Binding curves were obtained for 99.5 mol % POPC with 0.5 mol % NBD-PC as well as for 94.5 mol % POPC, 5 mol % POPS, and 0.5 mol % NBD-PC (Figure 4). The data with 99.5 mol % POPC gave rise to *K*_d_ = 0.83 μM ± 0.23, which is almost four times tighter than the corresponding measurement made in H_2_O. Moreover, the data with 5 mol % POPS shows a *K*_d_ value of 0.84 μM ± 0.17, which represented tightening by a factor of just over two compared to experiments in H_2_O. Both of these experiments corroborate the assumption that H-bonding plays a role in RuApy–bilayer interactions.

Another important point to explore in RuApy–lipid membrane affinity is the influence of hydrophobic interactions on the mechanism. This can be probed by introducing cholesterol into the membrane, which will decrease the area per lipid and therefore weaken interactions between RuApy and the hydrophobic core of the bilayer. To do this, 30 mol % cholesterol was introduced into membranes containing 69.7 mol % POPC and 0.3 mol % NBD-PC. Notably, the *K*_d_ value was 2.85 μM ± 0.70 (Figure S5), which is modestly tighter (less than a factor of 2) compared to the corresponding system without cholesterol. Because the affinity between RuApy and the membrane tightened slightly, the role of the hydrophobic tail region was probably not a major factor in the coordination complex-membrane interaction. Moreover, the slight enhancement is probably from the hydrogen bonding interaction between cholesterol and RuApy.

The thermodynamic data for the RuApy–membrane interactions considered above are summarized in Table 1. Taken together, these data suggest that H-bonding and electrostatic effects are more important for RuApy–membrane interactions than hydrophobic effects.

**Table 1:** *K*_d_ Values for RuApy/Phospholipids in SLBs

SLB composition (% mol)	buffer condition, pH 7.4	*K*_d_ (μM)
99.5% POPC, 0.5% NBD-PC	PBS	3.11 ± 1.11
	phosphate	0.22 ± 0.04
	PBS/D_2_O	0.83 ± 0.23
99.7% POPC, 0.3% TR-DHPE	PBS	3.6 ± 0.40
	phosphate	0.68 ± 0.12
94.5% POPC, 5% POPS, 0.5% NBD-PC	PBS	1.99 ± 0.29
	phosphate	0.089 ± 0.009
	PBS/D_2_O	0.84 ± 0.17
69.7% POPC, 30% cholesterol, 0.3% TR-DHPE	PBS	2.85 ± 0.70
94.5% POPC, 5% POPE, 0.5% NBD-PC	PBS	0.81 ± 0.05

### Surface Pressure–Area Isotherms in Langmuir Monolayers

**Figure 5:**
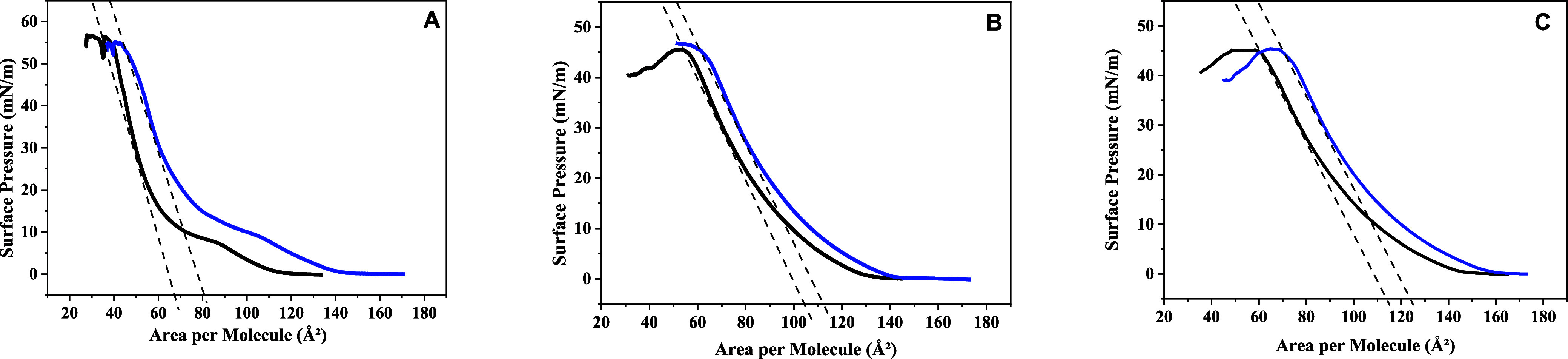
π–*A* isotherms in the absence (black data points and fits) and presence (blue data points and fits) of 2 μM of the RuApy complex for monolayers composed of (A) DPPC, (B) POPC, and (C) 95% POPC and 5% POPS.

Lipid area expansion information related to the introduction of RuApy could be obtained by performing surface pressure (π)–area (*A*) isotherm measurements with a Langmuir trough. Figure 5 shows π–*A* isotherms for (A) 1,2-dipalmitoyl-*sn*-glycero-3-phosphocholine (DPPC), (B) POPC, and (C) POPC + 5% POPS monolayers in PBS buffer in the absence (black data points) and presence (blue data points) of 2 μM RuApy in the subphase. In all three systems, the surface area per lipid increased at a given surface pressure upon introducing RuApy. The areas at 30 mN/m increased from 49 Å^2^ to 61 Å^2^ (Δ*A* = 13 Å^2^) for DPPC, from 70 Å^2^ to 78 Å^2^ (Δ*A* = 7 Å^2^) for POPC, and from 75 Å^2^ to 84 Å^2^ (Δ*A* = 10 Å^2^) for 95% POPC and 5% POPS. It should be noted that the data taken at 30 mN/m corresponds to the internal pressure of a lipid bilayer^29^ and therefore should correspond with the area per lipid in the supported bilayer measurements described above.

The increase in the area per lipid upon injection of RuApy into the subphase is consistent with the hypothesis of the coordination complex inserting between lipid headgroups and, thereby, expanding the monolayer. As expected, the area increase was the largest for DPPC because the acyl chains are saturated and initially closely packed in the gel phase. As such, the headgroups are close together, and the introduction of RuApy causes the largest area increase. For POPC, the area increase was slightly over half the value for DPPC. POPC has an unsaturation bond in the lipid tail, reflecting headgroups that are already further apart at a given pressure. RuApy, therefore, could be accommodated without as much of an expansion as observed for DPPC. The area increases in the monolayer with 5 mol % POPS were larger than for POPC but smaller than for DPPC. POPS should lead to more extensive hydrogen bonding with RuApy compared with pure POPC, which imposed greater restrictions on its orientation at the surface. This, in turn, caused greater expansion because the complex could not be easily reoriented to minimize the expansion. It should be noted that the differences in the collapse pressures observed between the monolayers formed of DPPC and both POPC and POPC/POPS mixtures can be attributed to the structural properties of their fatty acid chains. DPPC, with its saturated chains, forms more compact and ordered monolayers, resulting in a collapse pressure above 50 mN/m.^30,31^ In contrast, POPC, containing an unsaturated chain, leads to a less compact and more fluid monolayer, with a lower collapse pressure compared to DPPC monolayer, as previously reported.^32^ The addition of 5 mol % POPS, despite contributing a negative charge in the polar head moiety, does not significantly alter the fluidity of the monolayer, keeping the collapse pressure consistent with the presence of unsaturated POPC.

**Figure 6:**
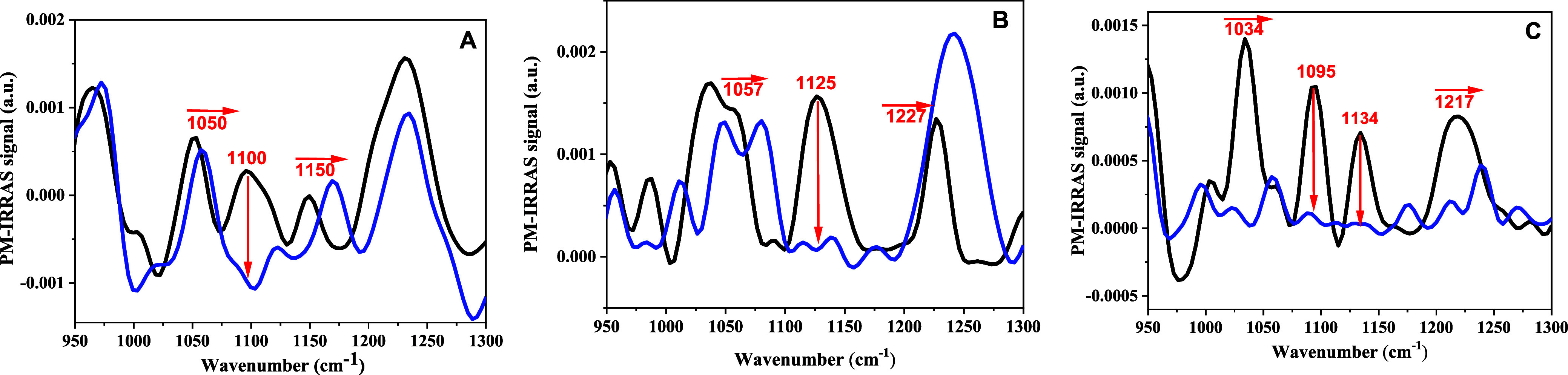
PM-IRRAS spectra of the headgroup region in the absence (black) and presence (blue) of 2 mM of RuApy in monolayers composed of (A) DPPC, (B) POPC, and (C) 95% POPC + 5% POPS.

The molecular-level interactions of RuApy with Langmuir monolayers composed of DPPC, POPC, and 95% POPC + 5% POPS were investigated by vibrational spectroscopy using PM-IRRAS. The spectra between 950 cm^–1^ and 1300 cm^–1^ are shown in Figure 6. The DPPC spectrum shows two peaks at 1100 and 1050 cm^–1^ assigned to the symmetric stretching of PO^2–^ (νsPO^2–^) and the phosphate ester stretching C-OP (ν C–OP), respectively.^33−36^ For POPC, the *v*(C–OP) absorption splits into two broad shoulders showing the third vibration assigned to the vibration of the R–O–P–O–R transition.^36^ The addition of POPS affects these transitions strongly, leading to one intense absorption at 1034 cm^–1^ with a weak shoulder at 1060 cm^–1^. This effect may be caused either by the disordered tail of POPS or by the negative charge on the headgroup. The 1100 cm^–1^ band due to ns(νsPO^2–^) almost completely disappeared in the presence of RuApy, likely caused by electrostatic interactions between the positively charged RuApy and the phosphate group. Similar results have been reported for metal ions.^33,37^ This interaction may cause changes in the ν(C–OP) and R–O–P–O–R vibrations. For DPPC, only a small change from 1050 to 1058 cm^–1^ is observed in the presence of RuApy. The corresponding absorptions for POPC become broader and split into two shoulders with RuApy. This change may be indicative of an increase in disorder of the hydrocarbon chains in POPC + POPS compared to pure POPC, in agreement with the π–*A* isotherm results. The incorporation of RuApy induces a blue shift in the 1150 cm^–1^ band of the choline moiety in DPPC. In a gel phase monolayer like DPPC, the P–N dipole is pointed almost straight up. When RuApy is introduced, the area per headgroup increases, as seen in the surface–area isotherm in Figure 5A. Hence, the P–N dipole should bend over for the choline of a lipid molecule to interact with the phosphate on an adjacent lipid. Indeed, the lack of a peak at 1150 cm^–1^ in the latter two cases (Figure 6B,C) helps to confirm the idea that the shift in (*A*) is in fact coming from P–N dipole reorientation.^38^

The position of the 1220 cm^–1^ peak associated with ν_as_(O=P=O) indicates that the phosphate moiety is highly hydrated in DPPC. When RuApy is introduced, there is only a modest shift; therefore, the phosphate moiety remains hydrated, and the P–N dipole still mostly remains oriented away from the plane of the bilayer. By contrast, in both Figure 6B and Figure 6C, a blue shift to 1240 cm^–1^ is apparent. This indicates substantial dehydration after the introduction of the coordination complex. The intensity of this peak increases in (B) but decreases in (C), which indicates differences in the reorientation of the phosphate dipoles in the two cases.

**Figure 7:**
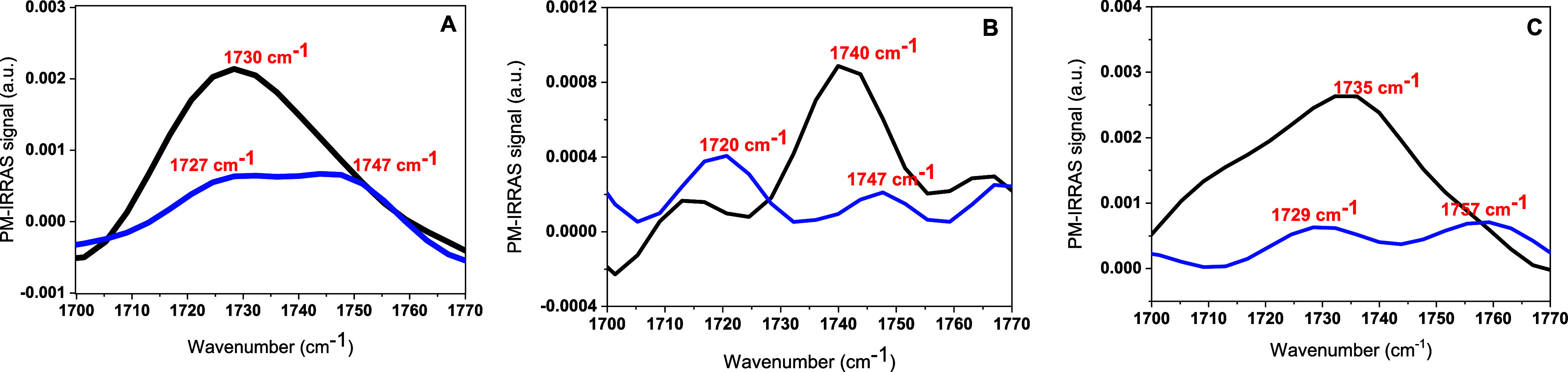
PM-IRRAS spectra for the carbonyl stretch region in the absence (black) and presence (blue) of 2 mM of RuApy for monolayers composed of (A) DPPC, (B) POPC, and (C) 95% POPC + 5% POPS.

The carbonyl band, C=O, which is located around 1730 cm^–1^ in the PM-IRRAS spectrum, can be used as a sensitive probe of hydration, polarity, and hydrogen-bonding in the region where the lipid headgroup meets the acyl chain region (Figure 7).^39^ In samples with a heterogeneous range of solvation environments, a broad band is observed between 1700 cm^–1^ and 1750 cm^–1^ region.^39−43^ This is precisely the case for DPPC in the absence of RuApy (Figure 7A). When RuApy is introduced, this peak is modestly attenuated and splits in two distinct contributions from the hydrogen bonded C=O at 1727 cm^–1^ and the non-hydrogen bonded mode at 1747 cm^–1^.^39,41^ By contrast, the data for POPC show two clearly resolved peaks even in the absence of RuApy (Figure 7B). The non-hydrogen bonded mode near 1740 cm^–1^ is dominant in this case. However, the introduction of RuApy causes the hydrogen bonded C=O band to become more pronounced at 1720 cm^–1^, while the 1747 cm^–1^ is sharply reduced. This case represents the strongest evidence of hydrogen bond formation in the ester region upon the introduction of RuApy. Finally, the system containing 5 mol % POPS in POPC shows an intermediate behavior, where the two bands become better resolved after the introduction of RuApy (Figure 7C). Probably, the evidence for hydrogen bonding is not clearly observed here because RuApy preferentially interacts with the amine and carboxylate on the PS lipids in this case rather than the phosphate moiety.

**Figure 8:**
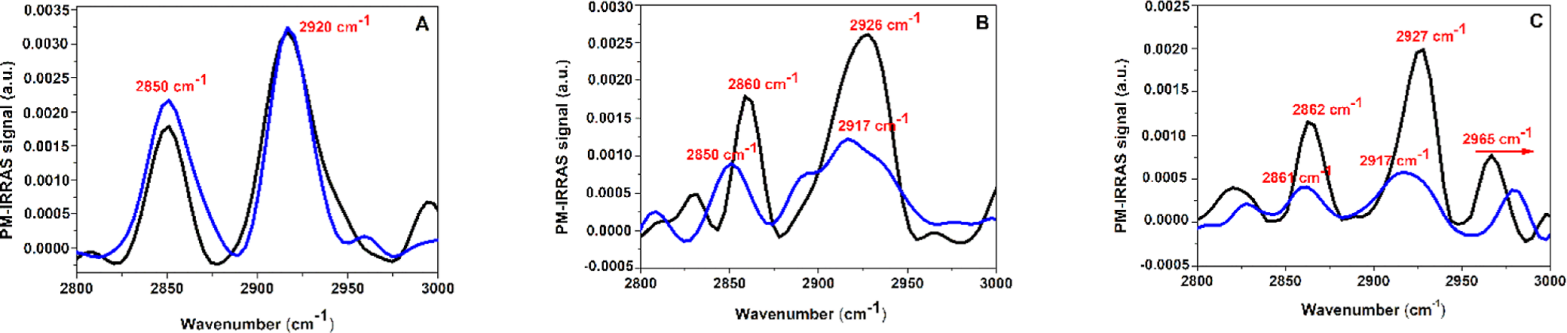
PM-IRRAS spectra for the alkyl region in the absence (black) and presence (blue) of 2 mM of RuApy for monolayers composed of (A) DPPC, (B) POPC, and (C) 95% POPC + 5% POPS.

The intensity ratio between the symmetric (2850 cm^–1^) and asymmetric (2920 cm^–1^) stretch modes of the CH_2_ groups (*I*ν_s_/*I*ν_as_) was employed to explore how RuApy influenced alkyl chain ordering·^44−46^ For the DPPC monolayer, this ratio increases from 0.56 to 0.66 when 2 μM RuApy was added to the aqueous subphase (Figure 8A). By contrast, these chains are already more disordered in the POPC monolayers even before the introduction of RuApy, whereby *I*ν_s_/*I*ν_as_ = 0.65, Figure 8B. This value increased to 0.74 when RuApy was added. This increase is consistent with a modest reduction in chain ordering at a constant surface pressure of 30 mN/m. For POPC + 5% PS, the variation of *I*ν_s_/*I*ν_as_ was similar to that found for DPPC (0.57–0.68), Figure 8C. The reason why the DPPC and 5 mol % POPS monolayers show approximately the same symmetric to asymmetric ratios may be related to the sites of unsaturation and the presence of hydrogen bonding in the second case. Nevertheless, the peak at 2965 cm^–1^ from the CH_3_ asymmetric stretch is much larger for the 5% POPS case, and the peak shifts upon adding RuApy, which is evidence of changes in ordering. By contrast, there is almost no 2965 cm^–1^ intensity for the DPPC system until RuApy is introduced. As such, this gel phase system is far better ordered, but it does show a modest amount of disordering once the complex is added.

Table 2 summarizes the assignments of the main vibrational bands in the PM-IRRAS spectra. The result is consistent with dehydration, altered hydrogen bonding, and changes in alkyl chain ordering upon adding RuApy. These effects vary with lipid composition. Specifically, the presence of PS lipids highlights the importance of electrostatic interactions in modulating changes.

**Table 2:** Assignment of the Bands in the PM-IRRAS Spectra in cm^–1^

	DPPC		POPC		95% POPC + 5% POPS	
bands assignment	PBS	RuApy	PBS	RuApy	PBS	RuApy
ν_as_ (CN^+^(CH_3_)_3_)	963	970	952	955	949	949
ν (C–OP)	1050	1058	1057	1080	1034	1060
ν_s_ (PO_2_)	1096		1125		1095	
ν_as_ (PO_2_)	1230	1233	1227	1240	1217	1240
ν (C=O)	1730	1727/1747	1740	1720/1747	1735	1729/1757
ν_s_ (CH_2_)	2850	2850	2858	2852	2864	2861
ν_as_ (CH_2_)	2916	2916	2927	2917	2926	2916

The combination of thermodynamic and spectroscopic experiments performed herein provides evidence that RuApy interacts with model lipid membranes mainly in the polar headgroup region of the lipids. These interactions are dominated by electrostatics and hydrogen bonding. Such interactions were in line with the significant changes in the dissociation constant of RuApy in the presence of anionic POPS lipids and by exchanging POPC for POPE as well as exchanging H_2_O for D_2_O. Furthermore, PM-IRRAS was able to identify the phosphate and carbonyl groups in the phospholipid bilayer which interacted with RuApy. These results were consistent with thermodynamic measurements using SLBs in the microfluidic channels.

## Conclusions

The relatively strong interactions of the positively charged RuApy with negatively charged membranes, such as those containing PS, are significant, given that amyloid β peptide (Aβ) can also bind to negatively charged lipid membranes, leading to pore formation and increased generation of toxic Aβ structures.

In this context, our findings provide a possible explanation for the protective effect of RuApy on PC12 cells against the toxic species associated with Aβ. Our results suggest that compounds able to shield the interactions of Aβ with lipid membranes could serve as a valuable tool in the development of antiamyloid drugs. Thus, positively charged compounds containing hydrophobic and hydrophilic regions in the structure, which can disturb the salt bridge interactions between Aβ and lipid membranes, could mitigate the toxic effects from Aβ.

## Experimental Section

### Materials

The solvents of HPLC grade used in synthesis and spectroscopic analysis of RuApy were purchased from Sigma-Aldrich and used as received without further purification. POPC, DPPC, POPE, POPS, and NBD-PC were purchased from Avanti Polar Lipids (Alabaster, AL). Cholesterol was purchased from Sigma-Aldrich. Texas Red 1,2-dihexadecanoyl-*sn*-glycero-3-phosphoethanolamine, triethylammonium salt (Texas Red DHPE) was purchased from Life Technologies (Grand Island, NY).

### Experimental Methods

The RuApy complex was prepared by reacting *cis*-[Ru(phen)_2_Cl_2_], previously prepared^47^ with 2 equiv of 3,4-aminopyridine in an H_2_O/EtOH (1:1) ratio using an experimental procedure already established by our research group.^48,49^ The complex was isolated as a hexafluorophosphate salt, and the structure was verified by ^1^H NMR (DMSO-*d*_6_) in a Bruker AVANCE III 600 MHz spectrometer, Figure S1: δ 9.45 (H1, H1′ dd), 8.85 (H3, H3′dd), 8.48 (H6, H6′ dd), 8.31 (H4,H4′ d), 8.27 (H2, H2′ m), 8.20 (H5, H5′ d), 7.97 (H8, H8′ dd), 7.58 (H7, H7′ m), 7.48 (a, a′ s), 7.31 (b, b′ d), 6.25 (c, c′ d), 5.88 (d, d′ s), 4.68 (e, e′ s). The ^1^H NMR spectrum shows duplication of the proton signals of 1,10-phenanthroline and 3,4Apy, consistent with two phen and two 3,4Apy ligands in the coordination sphere of Ru(II) in a *cis*-octahedral structure.

### Preparation of Small Unilamellar Vesicles

For small unilamellar vesicle (SUV) preparation,^50^ the lipids in the desired molar composition were well mixed in chloroform and then dried under a N_2_ stream to remove organic solvent. To remove any remaining solvent, the lipids were placed under vacuum for a minimum of 2 h. The lipid film was rehydrated with PBS, 10 mM, pH 7.4, to a concentration of 1 mg/mLl. Next, the suspensions were subjected to 10 freeze–thaw cycles using liquid nitrogen and a warm water bath. Afterward, the solution was extruded (10 mL LIPEX Extruder, Northern Lipids Inc., Vancouver, Canada) 10 times through a 100 nm pore polycarbonate membrane (Whatman, Florham Park, NJ), and the resulting vesicle solutions were stored at 4 °C.

### Glass Cleaning

The glass substrates used for supporting the lipid bilayers were made with Corning glass coverslips (24 × 40 mm, No. 1.5) and were cleaned with a 1:6 solution of 7× cleaning solution (MP Biomedicals, Solon, OH) and purified water, in which the coverslips were boiled for about 4 h to remove organic contaminants before they are annealed in a kiln. The coverslips were then rinsed with purified water before being dried thoroughly with nitrogen gas. This was repeated three times. Following this, the slides were annealed in a kiln (Sentry Xpress 2.0, Orton Ceramic Foundation, OH) at 500 °C for 5 h or overnight to make the glass surface smoother. Proper cleaning of glass is extremely important; if the glass coverslips are not properly prepared, then the diffusion constant and mobile fractions will be significantly worse, which will affect the homogeneity of the bilayer formed.^51−54^ The coverslips were stored in a clean box and kept in a clean room.

### Microfluidic Device Preparation

Microfluidic devices were fabricated using polydimethylsiloxane (PDMS) [Dow Corning Sylgard 184 Silicone Elastomer Kit, Ellsworth Adhesives (Germantown, WI)] and a template for the channels^55^ To make a microfluid device, PDMS was well mixed in a 10-part base to 1-part cross-linker mass ratio and put under vacuum for 2 h to eliminate the bubbles that were formed. Next, the PDMS was poured over the master slide template and placed in a 52 °C oven overnight. Outlets and inlets for each channel were punched using a hollow tube needle. The PDMS and glass slide were placed together in a plasma cleaner (PDC-32G, Harrick, Pleasantville, NY) and treated at 25 W with 53.2 Pa O_2_ for 45 s, and right after plasma cleaning, the glass slide and PDMS block were immediately pressed together, making the PDMS adhere to the cleaned glass coverslip, obtaining a microfluid device.

### Preparation of SLBs on Microfluid Channels

SLBs were formed in the microfluid channels via the vesicle fusion method,^56^ a widely used and reliable technique for creating homogeneous and continuous bilayers on solid surfaces.^57,58^ It is well-known by fluorescence recovery after photobleaching (FRAP) that membranes displayed uniform fluorescence intensity down to the diffraction limit, which suggests that bilayers are uniform.^59,60^ Thus, vesicle fusion has been extensively validated in various systems, ensuring the formation of defect-free bilayers with lateral diffusion properties that closely resemble those of natural membranes.^61^

Specifically, 10 μL of a mixture in a 1:1 proportion of the solution of vesicles (SUVs) previously prepared with a 500 mM NaCl solution was injected into the common outlet of the 8-channel device. Incubation was performed for approximately 10 min, and excess vesicles were rinsed away for more 10 min with pure PBS buffer. At the end of this process, the nascently formed bilayers inside the channels were ready for experiments.

### Fluorescence Microscopy

Fluorescence microscopy images of the SLBs were obtained with a Nikon Eclipse Ti–U fluorescence microscope (Tokyo, Japan) using a 10× objectives. The dye fluorescence was observed using different filter sets: Texas Red DHPE (λ_exc_ = 595 nm; λ_emi_ = 615 nm), 1-(palmitoyl-2-{12-[(7-nitro-2–1,3-benzoxadiazol-4-yl)amino]dodecanoyl}-*sn*-glycero-3-phosphocholine) NBD-PC (λ_exc_ = 470 nm; λ_emi_ = 530 nm), and RuApy (λ_exc_ = 480 nm; λ_emi_ = 655 nm). An exposure time of 500 ms was selected to minimize photobleaching but at the same time to give a sufficient fluorescence intensity response. Also, to avoid photochemical reactions with the ruthenium complex, the measurements were made in the dark, and only one image was taken for each condition.

### Dissociation Constant (*K*_d_) Measurements

The binding of RuApy to a SLB was monitored by the following procedures. First, fluorescence images of an SLB in the flow cell device were recorded in the absence of RuApy as well as after the addition of varying concentrations of RuApy. The dissociation constant was determined according to the methodology described in the literature.^5^ In this form, the data could be used to calculate the quenched fraction (QF) of dye-PC at each RuApy concentration (eq 1)
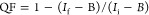
1*I*_f_ = normalized fluorescence concentration after the introduction of RuApy. *B* = background fluorescence intensity (i.e., between the channels). *I*_i_ = initial normalized fluorescence intensity.

By varying the complex concentration, it was possible to obtain a binding curve for the RuApy/membrane interaction (*K*_d_), and this data was fit to Langmuir isotherms (eq 2).^5^
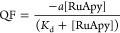
2

### Surface Pressure (π) vs Molecular Area (A) Isotherms (π–*A* Isotherms)

Experimental π–*A* isotherms were obtained by forming a Langmuir monolayer in a mini-KSV Langmuir trough (KSV Instruments, Helsinki, Finland) with an area of approximately 242 cm^2^ and a volume of 250 mL. The trough was equipped with a Wilhelmy sensor [filter paper (10 × 20 mm^2^)] to measure the surface pressure. For monolayer formation, 20 μL of the lipid solutions in chloroform at a concentration of 1 mg/mL was spread at the air/water interface with a Hamilton microsyringe, and the system was left standing for 10 min to allow complete evaporation of the chloroform.

Compression was performed at a barrier speed of 10 mm/s. Surface pressure/per unit of lipid molecule (π/*A*) isotherms provided information about the two-dimensional phases of the monolayer.

Infrared reflection–absorption spectroscopy (PM-IRRAS) measurements.

PM-IRRAS spectra were measured with a KSV PMI 550 instrument (KSV Instrument, Ltd.) that employs a silicon carbide lamp as an IR light source, a ZnSe photoelastic polarization modulator (PEM), and a HgCdTe detector (MCT) model PCI-3TE-10.6. The incident infrared beam is modulated by the PEM and then polarized in two planes (parallel, p and perpendicular, s to the incidence plane), focused at 80° relative to the normal of the subphase plane. The PM-IRRAS signal (*S*) is calculated using , where *R*_p_ is the reflectance at polarization p and *R*_s_ is the reflectance at polarization s. For this, the detector was placed above the mini-KSV Langmuir trough, where the monolayers were formed by compressing the lipids to a pressure of 30 mN/m, controlled via the Langmuir trough’s software, ensuring that the pressure was automatically maintained throughout the entire experiment. The positive or negative signals in PM-IRRAS are a characteristic feature of lipid monolayers at the air–water interface, resulting from the interaction of polarized light with the oriented molecular dipoles in the lipid film. Each spectrum was acquired through 6000 scans, resulting in a measurement duration of 10 min, maintaining the temperature at 22 ± 1 °C.

## Supporting Information Available

Materials and methods for the synthesis and characterization of RuApy and photophysical studies with RuApy and SLBs (PDF)
