# Supplementary material for: Binding of cis-[Ru(phen)2(3,4Apy)2]2+ to Model Lipid Membranes: Implications for New Tools in the Development of Antiamyloid Drugs
Source: Langmuir. 2024 Dec 17;40(52):27345–55. doi: 10.1021/acs.langmuir.4c03552 (PMC11697347; doi:10.1021/acs.langmuir.4c03552)
Supplement: Supplementary file 1 — la4c03552_si_001.pdf [file la4c03552_si_001.pdf]

Supporting Information

Binding of *cis*-[Ru(phen)$_2$(3,4Apy)$_2$]$^{2+}$ to model lipid membranes: Implications for new tools in the development of anti-amyloid drugs

Maria Laura a Cruz Garcia[†], *Rafaela Ribeiro Paixão*[†], *Wallance M. Pazin*[§], *Oswaldo N. Oliveira Jr.* [⊥]; *Paul S. Cremer*[‡*], *Rose Maria Carlos*[†*]

[†]. Department of Chemistry, Federal University of São Carlos, CP 676, CEP 13565-905, São Carlos-SP, Brazil

[§]. Department of Physics and Metereology, São Paulo State University, CEP 17033-360, Bauru – SP, Brazil

[⊥]. Sao Carlos Institute of Physics, University of Sao Paulo, CP 369, CEP 13560-970, São Carlos, SP, Brazil

[‡] Department of Chemistry and Department of Biochemistry and Molecular Cell Biology, The Pennsylvania State University, University Park, Pennsylvania 16802, United States

Table of Contents

Synthesis and characterization of RuApy complex	S3
Binding curves and K_d value for the RuApy/SLB	S5
References	S7

Synthesis and characterization of RuApy complex

The ^{1}H NMR spectrum of RuApy complex in DMSO-d_6 shows duplication of proton signals of 1,10-phenanthroline and 3,4-Apy in the coordination sphere of Ru(II) in a *cis*-octahedral structure, Figure S1.[1]

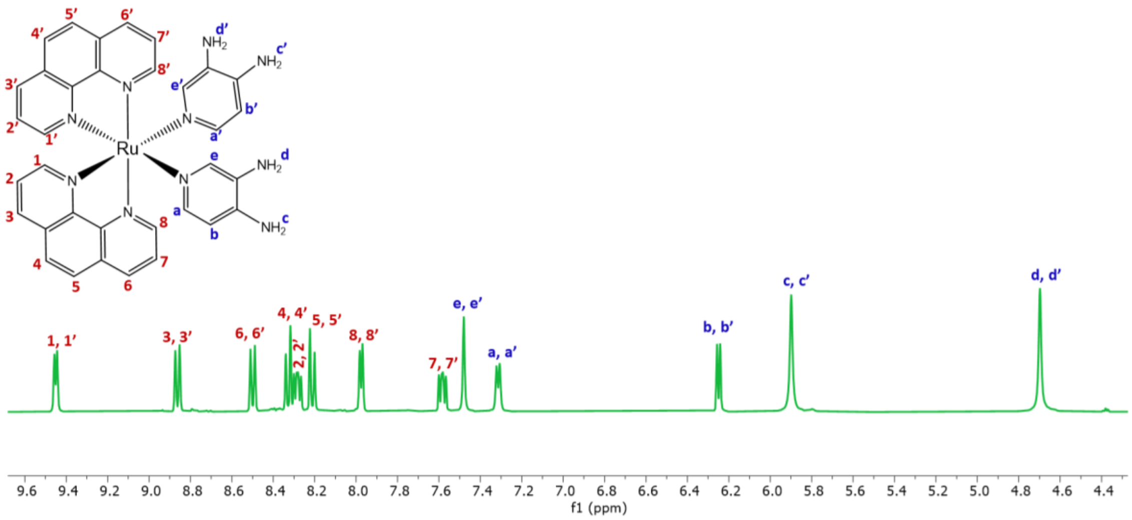

Figure S1. ^{1}H NMR of *cis*-$[Ru(phen)_2Apy_2](PF_6)_2$ in DMSO-d_6

Binding curves and K_d value for the RuApy/SLB

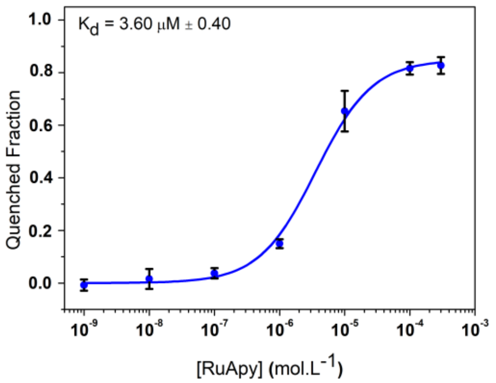

Figure S2. Curve obtained to determine K_d for RuApy/SLB interactions in PBS buffer 10 mM pH 7.4 137 mM of NaCl with bilayers composed of 99.**7**% POPC and 0.3% TR-DHPE.

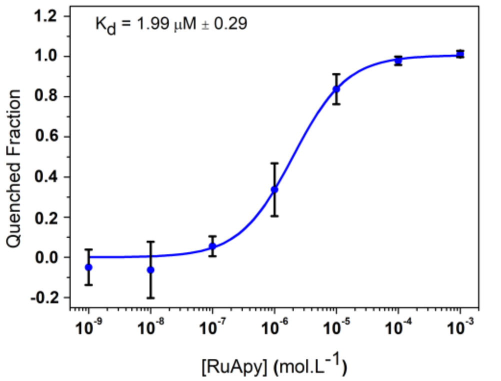

Figure S3. Curve obtained to determine K_d for RuApy/SLB interactions in PBS buffer 10 mM pH 7.4 137 mM of NaCl with bilayers composed of 94.5% POPC, 5% POPS and 0.5% NBD-PC.

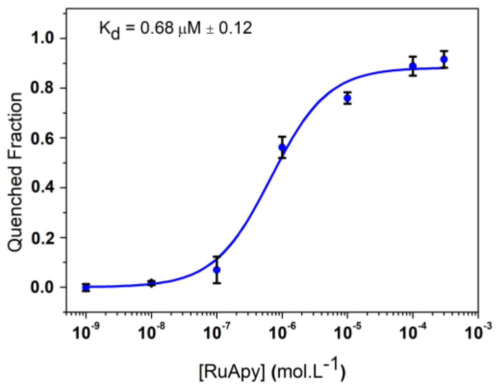

Figure S4. Curve obtained to determine K_d for RuApy/SLB interactions in Phosphate buffer 10 mM pH 7.4 without NaCl with bilayers composed of 99.7% POPC and 0.3% TR-DHPE.

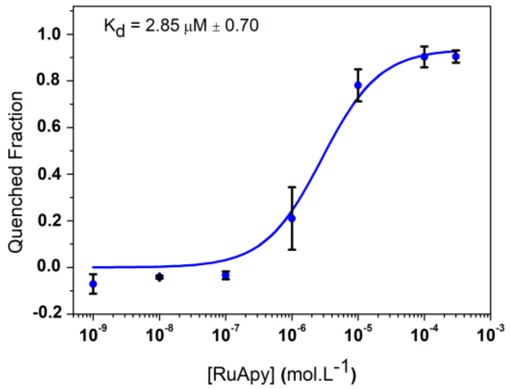

Figure S5. Curve obtained to determine K_d for RuApy/SLB interactions in PBS buffer 10 mM pH 7.4 with 137 mM of NaCl with bilayers composed of 69.7% POPC, 30% Cholesterol and 0.3% TR-DHPE.

REFERENCES

(1) Silva, D. E. S.; Cali, M. P.; Pazin, W. M.; Carlos-Lima, E.; Salles Trevisan, M. T.; Venâncio, T.; Arcisio-Miranda, M.; Ito, A. S.; Carlos, R. M. Luminescent Ru(II) Phenanthroline Complexes as a Probe for Real-Time Imaging of Aβ Self-Aggregation and Therapeutic Applications in Alzheimer's Disease. *J Med Chem* **2016**, *59* (19), 9215–9227. https://doi.org/10.1021/ACS.JMEDCHEM.6B01130/SUPPL_FILE/JM6B01130_SI_001.PDF.
